# Usefulness of Discarded Vitreous Samples from Routine Vitrectomy

**DOI:** 10.1155/2016/2380764

**Published:** 2016-04-26

**Authors:** Natàlia Vilà, Pablo Zoroquiain, Vasco Bravo-Filho, Emilia Antecka, Helena Dietrich, John C. Chen, I. John Galic, Michael A. Kapusta, Miguel N. Burnier

**Affiliations:** ^1^RI-MUHC, Henry C. Witelson Ocular Pathology Laboratory, 1001 Boulevard Decarie, Block E, Lab No. E02.2389, Montreal, QC, Canada H4A 3J1; ^2^Department of Ophthalmology, McGill University, Montreal, QC, Canada

## Abstract

*Purpose*. To describe the histopathological features of vitreous samples obtained after vitrectomy surgery from diabetic and nondiabetic patients.* Methods*. Vitreous specimens from 137 patients who underwent vitrectomy for different clinical conditions were analysed. All samples were centrifuged and each resulting pellet was fixed and processed as part of routine paraffin section histopathology. The histopathological features were categorized in a semiquantitative fashion. The samples from diabetic and nondiabetic patients were compared.* Results*. The 125 included patients (58 diabetic, 60% males) were aged 64.2 ± 13.9 years. The presence of hemorrhage, inflammatory cells, and histiocytes was significantly higher in the diabetic group (*P* < 0.001, *P* = 0.028, and *P* = 0.016, resp.), showing more vessels (*P* < 0.001) and ghost vessels (*P* = 0.049). The presence of inflammatory cells was the feature with the highest sensitivity for detecting diabetes mellitus (98%) and also the highest negative predictive value (89%). In the multivariate analysis, three variables emerged as independent significant predictors of diabetes in vitrectomy samples: hemorrhage, endothelial-lined vessels, and age (*P* < 0.001, *P* < 0.001, and *P* = 0.019, resp.).* Conclusions*. Different histopathological features can be found in vitreous samples from diabetic patients. Analysis of vitrectomy samples may serve as a tool for diabetes management.

## 1. Introduction

After more than four decades, pars plana vitrectomy has become routine for vitreoretinal surgeons. Vitrectomy can benefit patients with many ocular disorders such as retinal detachment, macular pathology, among other conditions. In addition, vitrectomy can be performed to assess vitreous cytology in order to rule out neoplastic processes in the clinical context of chronic nonspecific inflammation or masquerade syndrome [1]. When this differential diagnosis is not required, the material collected from vitrectomy surgery is frequently discarded because, to date, no importance has been attributed to these specimens.

The aim of the present study was to evaluate the histopathological features of vitreous samples in diabetic and nondiabetic patients. Diabetes mellitus (DM) is a metabolic disorder that affects vascular regulation and causes microvascular damage. Diabetic retinopathy (DR) is a common complication of diabetic microangiopathy, affecting about one-third of the patients with DM [2]. Moreover, prevalence increases with increased duration of DM [3]. Screening for DR is crucial for the early diagnosis of the asymptomatic clinical findings. At least, annual examinations of the ocular fundus are recommended for DR screening in diabetic patients [4].

Early stage of nonproliferative DR is manifested by excessive capillary permeability leading to inner blood retinal barrier dysfunction, capillary basement membrane thickening, pericyte and smooth muscle depletion, microaneurysm formation, capillary closure, and nonperfusion [5, 6]. Early signs of DR can only be detected by clinical observation when this whole process has already started. Despite new technologies to detect retinal changes [7], a methodology for early DR diagnosis before clinical and irreversible manifestations appear is not available.

To the best of our knowledge, no previous published reports have described the morphological features of diabetic vitreous cytology specimens.

## 2. Material and Methods

### 2.1. Vitreous Samples

Vitreous samples were collected from patients who underwent pars plana vitrectomy at Royal Victoria Hospital, Montreal, Quebec, Canada, from August 2012 to December 2012 for different clinical conditions (macular hole, epiretinal membrane, diabetic macular edema, pseudophakic cystic macular edema, vitreomacular traction syndrome, optic nerve pit, rhegmatogenous retinal detachment, tractional retinal detachment, vitreous hemorrhage, neovascular glaucoma, dislocated intraocular lens, traumatic cataract, retained lens fragment, or globe rupture). DM was established following the American Diabetes Association recommendations [8] according to data obtained from the patients' charts. Vitreous samples from diabetic and nondiabetic patients were included; the diabetic group included all patients with a DM diagnosis at the time of the procedure, irrespective of the presence of DR. Appropriate approval from the institutional review board was obtained. All data accumulation was in accordance with Canada and the Province of Quebec legislation and the tenets of the Declaration of Helsinki.

For vitrectomy processing, the entire contents of each cassette were processed. The samples were divided in 50 mL tubes and centrifuged at 2500 rpm for 15 minutes after which the supernatant was discarded. All pellets were then resuspended, mixed, and recentrifuged until one single pellet was collected. The resulting pellet from each sample was fixed in a 1 : 1 alcohol and formalin solution. The supernatant was poured off and the sediment was processed as part of routine paraffin section histopathology. The slides were stained with hematoxylin and eosin and scanned using a digital slide scanner (Aperio ScanScope AT Turbo, Leica Microsystems Inc., Concord, ON, CA). Slides that did not contain sample were excluded from further evaluation.

### 2.2. Morphological Evaluation

Histopathologically, all samples were evaluated according to the following findings: (1) sample characteristics: amount of sample was semiquantitatively evaluated (1 = low; 2 = high) and type of matrix was recorded as serous type (=1) or proteinaceous type (=2) when proteinaceous material (colloid like structures) was found; (2) the number of the following cells which was evaluated semiquantitatively (0 = negative; 1 = low; 2 = high): spindle cells (all cells with a spindle shape), retinal pigment epithelium cells (RPEc), histiocytes, inflammatory cells (including lymphocytes and neutrophils), and erythrocytes (hemorrhage); (3) vascular findings: the presence of vessels was analysed semiquantitatively (0 = negative; 1 = low; 2 = high) and classified as endothelial-lined, which could be subclassified into aneurismatic when the lumen was dilated or as a ghost vessel when no endothelium was present.

### 2.3. Statistical Analysis

Baseline characteristics in both groups were compared using the chi-square test or Fisher's exact test, when required. All features were analysed qualitatively (presence or absence) and semiquantitatively (negative, low, or high). The positive predictive value (PPV), negative predictive value (NVP), sensitivity or true positive (TRP), and specificity (SPC) were calculated for the features with *P* values ≤ 0.05. For each of the nine histopathological variables included in the analysis, a multivariate logistic regression model using a forward stepwise analysis was attempted to determine possible features that predict the diabetic condition. In this model, hypothetical confounding variables including age, amount of material, and gender were considered. All analyses were performed with* SPSS Statistics v. 21* (IBM Corp., Armonk, NY, USA). *P* values ≤ 0.05 were considered significant.

## 3. Results

Samples from 137 patients were initially analysed. However, 12 (8.7%) samples were excluded due to scant material on the slides. Therefore, samples from 125 out of 137 (91.3%) patients were finally included. Sixty per cent (*n* = 75) were male and 40% (*n* = 50) were female. The mean age of the included patients was 64.2 ± 13.9 years. Overall, 46.5% of the patients were diabetic, while the remaining 53.5% were nondiabetic. The mean age of the diabetic group was 59.6 ± 13.7 and the nondiabetic group 68.2 ± 12.9, which was significantly different (*P* < 0.001).

**Figure 1:**
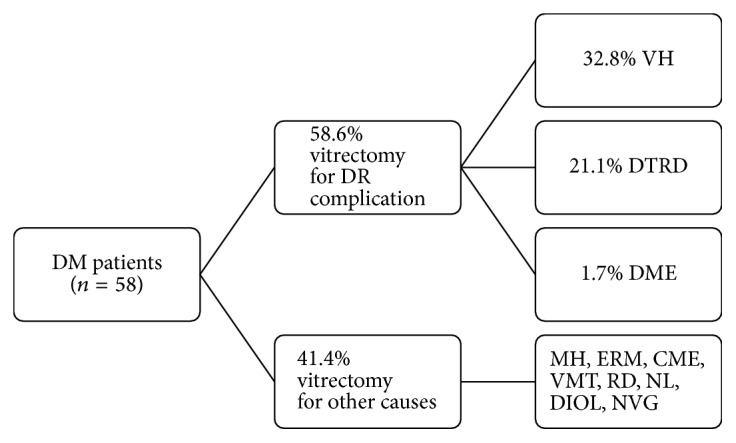
Underlying conditions for requiring a vitrectomy in diabetic patients. DM: diabetes mellitus; PPV: pars plana vitrectomy; DR: diabetic retinopathy; VH: vitreous hemorrhage; DTRD: diabetic tractional retinal detachment; DME: diabetic macular edema; MH: macular hole; ERM: epiretinal membrane; CME: cystoid macular edema; VMT: vitreomacular traction; RD: retinal detachment; NL: nucleus loss; DIOL: dislocated intraocular lens; NVG: neovascular glaucoma.

In the diabetic group, the most frequent diagnosis prior to surgery was vitreous hemorrhage (VH); however, 15.8% of these patients did not have proliferative DR as the underlying cause of the VH. Overall, 41.4% of DM patients underwent vitrectomy for other clinical conditions not related to DR. Proliferative DR (PDR) was present in 53.4% of diabetic patients (Figure 1). In the nondiabetic group, the most common underlying conditions were retinal detachment and epiretinal membranes. In this group, only 4.5% of the patients had a clinical diagnosis of VH (Table 1).

### 3.1. Samples

**Figure 2:**
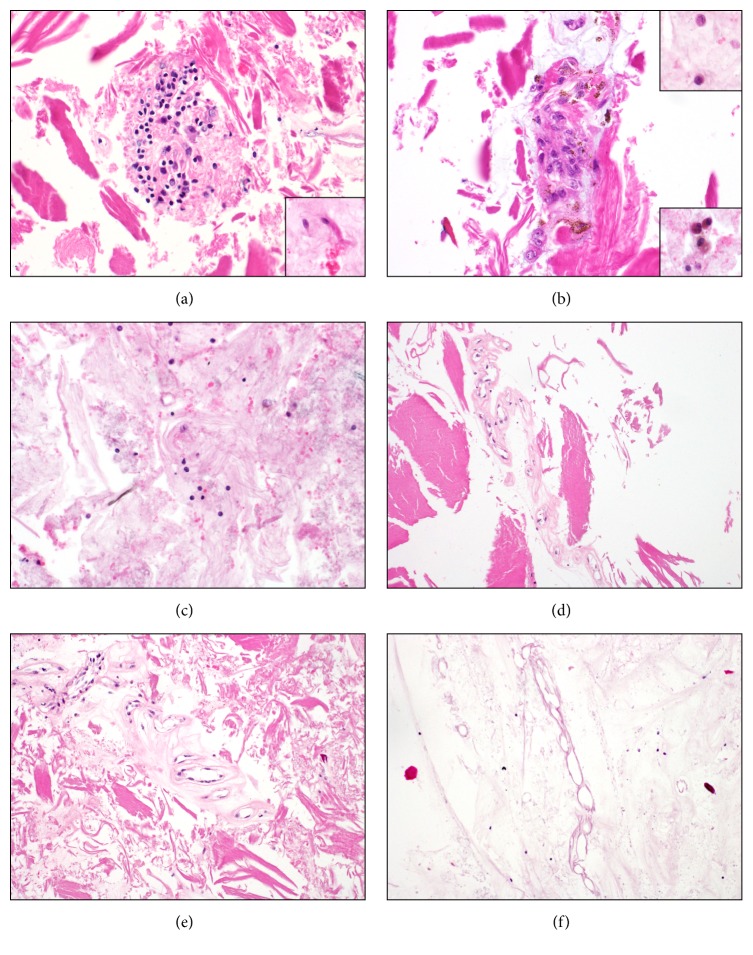
(a) Fragments of a group of spindle cells in type 2 matrix background. Inset shows isolated spindle cells (H&E, 40x). (b) Group of histiocytes and RPE cells in a granulomatous-like structure. Superior inset: isolated macrophages; inferior inset: isolated RPE cells (H&E, 40x). (c) Lymphocytes, neutrophils, and erythrocytes in type 1 matrix background (H&E, 40x). (d) Blood vessels with endothelial cell lining (H&E, 20x). (e) Aneurismatic dilation of a vessel (H&E, 20x). (f) Blood vessel without endothelial lining (ghost vessels; H&E, 20x).

In total, 100% of the histopathological features described in the methodology were morphologically identifiable (Figure 2). The amount of sample processed and the type of matrix did not show differences between diabetic and nondiabetic samples.

### 3.2. Types of Cells

In the qualitative analysis, the presence of histiocytes, inflammatory cells, and erythrocytes was significantly higher in the diabetic group than in the nondiabetic group (*P* = 0.016, *P* = 0.028, and *P* < 0.001, resp.). However, in the semiquantitative analysis only the erythrocytes showed differences being higher in the diabetic group (*P* < 0.001). The presence of spindle and RPEc showed no differences between groups, both qualitatively (*P* = 0.403 and *P* = 0.102, resp.) and semiquantitatively (*P* = 0.067 and *P* = 0.159, resp.).

### 3.3. Vascular Findings

Aneurismatic dilatation did not show significant differences between groups. We observed only 4 (6.9%) diabetic patients with aneurismatic dilatation. In the diabetic group, 16 patients (27.6%) had ghost vessels whereas only 9 (13.4%) did in the nondiabetic group, this difference being statistically significant (*P* = 0.040). The presence of endothelial cell-lined vessels was higher in the diabetic group both qualitatively and semiquantitatively (*P* < 0.001 and *P* < 0.001, resp.) (Table 2).

### 3.4. Statistical Analysis

The presence of inflammatory cells was the feature with the highest TRP for detecting DM (98%) and also the highest NPV (89%). The endothelial-lined vessels showed the highest SPC (90%) and PPV (76%). The combination of three features in the same sample such as inflammatory cells, endothelial-lined vessels, and hemorrhage revealed a PPV of 83%, NPV of 100%, TRP of 100%, and SPC of 80%.

In the multivariate logistic regression we tested the relationship between DM diagnosis and eight independent histopathological variables (spindle and inflammatory cells, RPEc, histiocytes, erythrocytes, aneurismatic dilatation, endothelial-lined vessels, and ghost vessels), adjusted by age, gender, and amount of material. Three variables emerged as independent significant predictors of diabetes in vitrectomy samples: hemorrhage, endothelial-lined vessels, and age (*P* < 0.001, *P* < 0.001, and *P* = 0.019, resp.). When considering variables semiquantitatively, only hemorrhage showed a correlation with risk of DM diagnosis. Higher numbers of erythrocytes in a vitrectomy sample (score of 2) showed an odds ratio (OR) of 13.4 (*P* < 0.001; 95% confidence interval [CI] 3.91–46.2; Table 3). However, low presence of erythrocytes (score of 1) was not a significant predictor. In this particular model, a greater age played a role as a protective predictor (*P* = 0.019; 95% CI 0.931–0.994) (Table 3).

## 4. Discussion

This study shows that vitreous cellblocks obtained from vitrectomy surgery can be used for histopathological analyses. Not only can enough material be obtained, but also differences between diabetic and nondiabetic patients are revealed in the histopathological features of their vitreous samples. The presence of hemorrhage, histiocytes, inflammatory cells, vessels, and ghost vessels was associated with a DM diagnosis. In addition, some features were significantly higher in specimens from diabetic patients even if they had not been diagnosed with DR at the time of the surgical procedure.

The technique used for our analyses, a modification of the classical cellblock technique [9], has shown high efficacy—useable samples were generated in 91.3% of cases—and, more interestingly, it allows the use of material from vitrectomy cassettes, which are usually discarded after surgery.

In diabetic patients, the histopathological findings were consistent with the expected results in patients with clinical signs of DR. This fact leads us to believe that the methodology used in this study would also allow us to obtain valid cytological samples from patients with other ocular conditions where a vitrectomy surgery might be performed.

The gold standard test for DM diagnosis is a simple laboratory test. In our study, when three specific characteristics were simultaneously detected in one sample (inflammatory cells, hemorrhage, and endothelial-lined vessels), the TRP and SCP values were close to the ideal screening test (TRP: 100%, SPC: 80%). A surgical procedure should not be used for screening purposes, but it can be useful in different scenarios: (1) patients with unknown DM who undergo vitrectomy surgery for any clinical condition in whom these characteristics are found (DM diagnosis should be confirmed) and more interestingly (2) patients with known DM but no clinical signs of DR who undergo vitrectomy with histopathological findings consistent with DM. These morphological features could be useful to stratify DR in early stages before the clinical diagnosis is made and help in the detection of subclinical manifestations. Besides, these patients should be further evaluated because stricter-than-typical metabolic control might delay the clinical manifestation of DR.

The pathophysiology of the DM can explain the presence of the specific cell types found differently expressed between groups. The presence of hemorrhage, histiocytes, inflammatory cells, vessels, and ghost vessels was associated with a DM diagnosis.

The presence of hemorrhage was significantly higher in the diabetic group. This was an expected result because 31%–54% of the VH are associated with DR [10]. The presence of abnormal vessels has shown spontaneous bleeding tendencies in PDR, which explains the presence of blood cells in the diabetic group. Fifty-seven per cent of the patients in the diabetic group with hemorrhage were categorized as “high,” and 57.9% of these patients were diagnosed with PDR. Conversely, 14 out of 67 patients (20.9%) in the nondiabetic group showed vitreous hemorrhage and only in four patients (28.6%) was hemorrhage categorized as “high” (67.0% presented with posterior vitreous detachment and a retinal tear). All the vascular features that were higher in diabetic patients (vessels with endothelium and ghost vessels) correlate as well with DR [11, 12].

In the diabetic group, all specimens (100%) presented inflammatory cells, which was not always related to the presence of hemorrhage because only 55.4% presented associated VH. The absence of inflammatory cells could be a marker of nondiabetic condition because this feature presented a NPV of 89%. The TRP and SPC were 98% and 12%, respectively. The low SPC could be explained by the fact that these cells are related to most inflammatory responses (acute or chronic), and almost all disorders that require surgery have a secondary inflammatory process (including trauma, VH, and retinal detachment). Further studies analysing the subpopulation of lymphocytes (B versus T and helper or cytotoxic) may provide more specific information.

In addition, histiocytes differed between groups; the higher presence in diabetic patients can be explained because of the frequent presence of histiocytes under chronic inflammatory conditions, such as DM.

The inherent limitations of a retrospective study are well known. A healthy control group was not available because vitrectomies are not performed usually in healthy eyes. The floaterectomy, or vitrectomy due to floaters, which is not a common practice in a socialized medical health system such as the Canadian, could be the ideal control group in future studies. Although the postoperative diagnosis was not available in a small number of final surgical reports in the digital file system, the patients were included in the final analysis as the preoperative diagnosis and the diabetic condition were reported.

The mean age of both groups was significantly different, the diabetic group being younger. In our particular model, the fact that 30% of the DM patients who underwent vitrectomy secondary to DM complication were younger than 50 years of age might be the underlying cause of this finding. It should be also considered that 43% of young patients with type 1 DM will develop PDR [13], which increases the chances of undergoing surgery and biases the age in this group.

Despite the differences encountered between groups, the only two independent predictive variables of DM in our multivariate analysis were the presence of hemorrhage and endothelial-lined vessels. Future prospective studies with a larger number of samples might reveal other histopathological features, which could play a role in predicting DR.

Further studies to detect early specific diabetic events would help to better determine the importance of the cytological features of DM and DR. The correlation between these vitreous morphological findings, systemic clinical information, and DR stage could provide the clinicians more information regarding DM manifestations and DR.

## 5. Conclusions

In summary, samples obtained from the vitrectomy machine cassette, which are routinely discarded, have enough tissue to identify histopathological findings related to DM and provide valuable information. Our methodology can be extrapolated in further studies to detect if preclinical stages of DR can be identified and also to add an extra tool for patients' management.

## Figures and Tables

**Table 1:** Baseline characteristics and postoperative diagnosis.

	Diabetic (*n* = 58)	Nondiabetic (*n* = 67)	Total (*n* = 125)	*P* value
Baseline characteristics				
Male, *n* (%)	37 (63.8)	38 (56.7)	75 (60)	NS
Age (mean ± SD)	59.6 ± 13.7	68.2 ± 12.9	64.2 ± 13.9	<0.001

Clinical diagnosis, *n* (%)				
Macular hole	1 (1.7%)	9 (13.4%)	10 (8%)	0.037
Epiretinal membrane	2 (3.4%)	15 (22.4%)	17 (13.6%)	0.002
Diabetic macular edema	1 (1.7%)	0 (0.0%)	1 (0.8%)	NS
Pseudophakic cystic macular edema	0 (0.0%)	1 (1.5%)	1 (0.8%)	NS
Vitreomacular traction syndrome	1 (1.7%)	0 (0.0%)	1 (0.8%)	NS
Optic nerve pit	0 (0.0%)	1 (1.5%)	1 (0.8%)	NS
Rhegmatogenous retinal detachment	5 (8.6%)	15 (22.4%)	20 (16.0%)	0.036
Tractional retinal detachment	0 (0.0%)	1 (1.5%)	1 (0.8%)	NS
Diabetic tractional retinal detachment	14 (24.1%)	0 (0.0%)	14 (11.2%)	<0.001
Vitreous hemorrhage	19 (32.8%)	3 (4.5%)	22 (17.6%)	<0.001
Retained silicone oil	0 (0.0%)	1 (1.5%)	1 (0.8%)	NS
Neovascular glaucoma	1 (1.7%)	0 (0.0%)	1 (0.8%)	NS
Dislocated intraocular lens	1 (1.7%)	1 (1.5%)	2 (1.6%)	NS
Traumatic cataract	0 (0.0%)	2 (3.0%)	2 (1.6%)	NS
Nucleus loss	3 (5.2%)	5 (7.5%)	8 (6.4%)	NS
Globe rupture	0 (0.0%)	2 (3.0%)	2 (1.6%)	NS
Nonavailable	10 (17.2%)	11 (16.4%)	21 (16.8%)	NS

SD: standard deviation; NS: nonsignificant.

**Table 2:** Characteristics of the diabetic and nondiabetic vitrectomy samples.

	Nondiabetic				Diabetic				*P* value	
	Absence/negative	Low	High	Total presence	Absence/negative	Low	High	Total presence	1	2
Sample characteristics										
Material	0	31	36	67	0	21	37	58	NS	—
Matrix	0	35	32	67	0	29	29	58	NS	—

Cell type										
Spindle cells	13	42	12	54	8	29	21	50	NS	NS
Retinal pigment epithelial cells	34	13	20	33	21	19	18	37	NS	NS
Histiocytes	30	21	16	37	14	24	20	44	NS	0.016
Inflammatory cells	8	53	6	59	1	48	9	57	NS	0.028
Erythrocytes	53	10	4	14	27	12	19	31	<0.001	<0.001

Vascular findings										
Vessels with endothelium	60	7	0	7	33	14	11	25	<0.001	<0.001
Aneurismatic dilatation	66	1	0	1	54	3	1	4	NS	NS
Ghost vessels	58	5	4	9	42	8	8	16	NS	0.049

*P* value 1: stratified quantitative analysis; *P* value 2: qualitative analysis (presence or absence); NS: nonsignificant.

**Table 3:** Multivariate logistic regression analysis of diabetes mellitus in vitreous samples, estimation of odds ratio in the presence of different histopathological features.

Multivariate model		
	OR (95% CI)	*P* value
Histopathological features		
Spindle cells	0.499 (0.140–1.781)	NS
Retinal pigment epithelial cells	0.810 (0.297–2.212)	NS
Histiocytes	1.423 (0.481–4.208)	NS
Inflammatory cells	6.055 (0.384–95.478)	NS
Erythrocytes	4.976 (2.008–12.348)	0.001
Endothelial-lined vessels	7.267 (2.352–22.448)	0.001
Aneurismatic dilatation	0.825 (0.065–10.471)	NS
Ghost vessels	1.423 (0.456–4.561)	NS

OR: odds ratio; CI: confidence interval; NS: nonsignificant.
